# Human–Machine Differentiation in Speed and Separation Monitoring for Improved Efficiency in Human–Robot Collaboration

**DOI:** 10.3390/s21217144

**Published:** 2021-10-28

**Authors:** Urban B. Himmelsbach, Thomas M. Wendt, Nikolai Hangst, Philipp Gawron, Lukas Stiglmeier

**Affiliations:** Work-Life Robotics Laboratory, Department of Business and Industrial Engineering, Offenburg University of Applied Sciences, 77723 Gengenbach, Germany; thomas.wendt@hs-offenburg.de (T.M.W.); nikolai.hangst@hs-offenburg.de (N.H.); philipp.gawron@hs-offenburg.de (P.G.); lukas.stiglmeier@hs-offenburg.de (L.S.)

**Keywords:** human–robot collaboration, speed and separation monitoring, human–machine differentiation, thermal cameras, protective separation distance

## Abstract

Human–robot collaborative applications have been receiving increasing attention in industrial applications. The efficiency of the applications is often quite low compared to traditional robotic applications without human interaction. Especially for applications that use speed and separation monitoring, there is potential to increase the efficiency with a cost-effective and easy to implement method. In this paper, we proposed to add human–machine differentiation to the speed and separation monitoring in human–robot collaborative applications. The formula for the protective separation distance was extended with a variable for the kind of object that approaches the robot. Different sensors for differentiation of human and non-human objects are presented. Thermal cameras are used to take measurements in a proof of concept. Through differentiation of human and non-human objects, it is possible to decrease the protective separation distance between the robot and the object and therefore increase the overall efficiency of the collaborative application.

## 1. Introduction

Human–Robot Collaboration (HRC) is seeing an enormous growth in research interest as well as in industry applications. The highest priority in HRC applications is given to the safety of the human within the system. A human within a robotic system is called an operator. Different approaches on how to protect the operator from any harm are subject to research. There has been good progress on how to protect the operator from any harm. The efficiency of the systems suffered from most of these safety improvements. Reduced efficiency leads to a reduced acceptance of HRC. In order to increase the acceptance, it is important to examine how these methods for operator safety can become more efficient.

Operator safety does not necessarily mean preventing the operator only from any physical contact. It can also mean to prevent psychological harm through dangerous and threatening movement of the manipulator. An overview of different methods of safe human–robot interaction can be found in [1]. Lasota et al. divided their work into four major categories of safe HRC: safety through control, through motion planning, through prediction, and through psychological consideration. The category of safety through control is subdivided into pre- and post-collision methods [1].

Speed and separation monitoring (SSM), which is subject of this work, belongs to the subcategory of pre-collision methods. Other methods of this subcategory are quantitative limits and the potential field method [2].

Established methods for HRC have already been integrated into standards like the ISO/TS 15066. The Technical Specification 15066 differentiates between four different modes of collaborative operations [3]:

- safety-rated monitored stop (SRMS),
- hand guiding (HG),
- speed and separation monitoring (SSM), and
- power and force limiting (PFL).

This paper focuses on Speed and Separation Monitoring (SSM). There are different sensor systems that can detect the Separation and Speed between the robot and the operator.

There are already quite a few well working sensor systems for speed and separation monitoring on the market. One can distinguish these systems as external and internal. Internal means that the sensors are part of the robot itself, e.g., mounted somewhere on the manipulator surface. External means that the sensor is placed on the edge of the table that the robot is mounted on, or on ceiling above the robot’s workspace. Examples for external sensor systems are laserscanners like [4], camera systems like the SafetyEYE [5] or pressure-sensitive floors [6]. There are only few examples for sensor systems that are mounted on the manipulator itself. A good example is the Bosch APAS system [7]. It consists of a safety skin that measures the separation distances capacitively. The main disadvantage is that it can only detect an obstacle in a distance of two to five centimeters.

All of these systems are more or less great in detecting obstacles within the workspace. It is difficult for them—if not impossible—to classify the obstacles in human and non-human objects. Non-human objects, like an automated guided vehicle (AGV), are therefore treated like an operator and safety measures are applied accordingly when they enter or pass through the workspace and its surroundings. These AGVs have fixed and well known dimensions. They have the ability to be programmed for certain behaviour and usually have a navigation system. Therefore, it should be possible to integrate an AGV with high precision into the robotic system for example in order to deliver and pick up workpieces. If the AGV is part of the entire system, it should not be treated as an operator. Instead, it should be possible to continue the robot’s movement with high velocities and consequently increase the overall efficiency of the system.

A good overview on research of concepts and performance of SSM can be found in [8,9]. Lucci et al. proposed in [10] to combine speed and separation monitoring with power and force limiting. This way it is possible to continue the movement of the robot when the operator is very close to the robot. A complete halt of the robot’s motion is only necessary when it comes to a contact between the operator and the robot. They showed that with their approach it is possible to increase the overall production efficiency. Kumar et al. researched on how to calculate the amount of sensors needed for a specific area as well as how SSM can be achieved from the surface of the robot [11,12]. Grushko et al. proposed the approach of giving haptic feedback to the operator through vibration on the operator’s work gloves [13]. The system monitors the workspace with three RGB-D cameras. The controller calculates if the operator’s hand intersects with the planned path of the robot and gives appropriate feedback to the operator. They were able to proof in user studies that the participants could finish their task more efficiently compared to the original baseline. A trajectory planning approach was taken by Palleschi et al. in [14]. Using a visual perception system to gather position data of the operator, they also used an interaction/collision model from Haddadin et al. [15] to permanently check the safety situation according to the ISO/TS 15066 standard. If the safety evaluation showed that the robot needs to slow down, their algorithm searched for an alternative path with lower risk for injury and velocities acceptable according to the safety limits. In an experimental validation the group was able to proof the effectiveness of planning safe trajectories for a task of unwrapping an object. Another approach based on dynamically scaled safety zones was proposed by Scalera et al. in [16]. Bounding Volumes around the robot links and the operator’s body and extremities represent the safety zones. These safety zones vary in their size according to the velocities of the robot and operator. Information about the operator’s position is gathered through a Microsoft Kinect camera. The paper proofed in a collaborative sorting operation that it was possible to shorten the task completion time by 10%.

As there is no system available that can distinguish between human and non-human objects, we see demand for such a system and the need to research methods on how to integrate the differentiation of human and non-human objects in existing safety methods.

In this paper, we propose a method on how such a differentiation is possible. Different sensor principles are presented that are capable of differentiating between human and non-human objects. Thermal cameras with two different field of views (FoV) are used to make measurements. The results show that it is possible two detect an operator in ranges up to 4 m from the sensor.

We have shown in previous publications that it is possible to do speed and separation monitoring directly from the robot arm. We showed that time-of-flight (ToF) sensors are suitable for this task. The first approaches used a camera mounted on the flange of the robot [17]. Further research investigated the use of single-pixel ToF sensors distributed over the links of the manipulator [18]. In [19], we presented a sensor solution in form of an adapter-plate that is mounted between the flange of the robot and the gripper. The previous work also showed that there is still potential for further growth of HRC applications in industrial settings. It also showed that the efficiency will play a key role in the success of HRC and that there is a need to improve the efficiency of these systems.

This paper proposes to differentiate obstacles in the vicinity of robotic systems into human and non-human objects. With this classification, it is possible to calculate object specific distance and velocity limits for the robotic system. The limits for non-human objects can be lower because there is only a financial risk associated with it instead of possible injuries to the human. As a result, it is possible to increase the efficiency of human–robot collaborative applications. The paper shows that a differentiation in certain applications is possible with thermal cameras that can be attached to manipulator or gripper. There is no need for an additional camera system surrounding the robot’s workspace and there is no need for any equipment to be attached to objects that shall be differentiated. The main contributions of this work can be summarized as shown in Table 1.

The paper is structured as follows. Section 1 introduces the topic and the state of the art. Section 2 gives an overview of sensor systems that can be used for human–machine differentiation in the context of speed and separation monitoring in human–robot collaboration. In Section 3 the protective separation distance is explained and the new object-specific protective separation distances is proposed. Furthermore, this section shows the potential efficiency improvement that can be established with this method. Section 4 explains what kind of and how the measurements were executed. Section 5 shows and discusses the results of the measurements before Section 6 concludes the paper and gives an outlook on future research.

## 2. Methods for Human–Machine Differentiation

There are active and passive methods to differentiate between human and non-human objects. Active methods are, for example, when camera systems are used and the AGV is marked with a sticker or QR-Code that identifies the object. Other active methods would be when the AGV sends its coordinates via wireless communication to the robotic system so that the robotic system knows exactly where the AGV is located and can therefore differentiate the AGV from other objects in the surroundings. A list of examples for active and passive methods is given in Table 2. The same is true for humans; they could wear a kind of tracker to monitor their position and send it to the robotic system. Depending on the overall situation on industrial shop floors, there might already exist a navigation system that keeps track of all machines, AGVs, and operators.

There are many small- and medium-sized enterprises (SMEs) that are new to automation with collaborative robots. They usually do not have any existing navigation or monitoring systems. Moreover, they require flexible solutions. Passive differentiation methods provide the most flexibility. They do not require any additional installations in the surroundings, on the operator or the AGV. This paper focuses on passive differentiation methods that will be presented in the following subsections.

These passive methods use sensors that measure properties that are characteristic for humans or machines. Here is a list of human specific properties that can be used for differentiation [20]:

- Temperature
- Size
- Weight
- Number of legs
- Heart Beat
- Breath

Depending on the specific property that needs to measured, the sensors can be placed at different locations. Three different locations are proposed that make sense to place the sensors. These are the base of the robot, the robot links itself, and the flange or gripper. For integration of the sensors in the gripper, a very flexible method is to use 3D printed grippers. Using 3D printing technology, it is possible to arrange and layout the sensors as needed. A good overview on this topic can be found in [21]. The following sections describe some possible sensor principles that can be used for passive human–machine differentiation.

### 2.1. Pressure-Sensitive Floor

If the mass of the automated guided vehicle (AGV) is known, and if this mass is different to the mass of the operators working around the robotic system, then it should be possible to differentiate between human and non-human objects by the difference in their mass. An improved system might be able to detect whether the object has two feet on the ground or if there are four wheels touching the ground. AGVs might have a different and more consistent footprint on the pressure sensitive floor. A human being has a variation in pressure. While walking, the human lifts up one foot and there is only one foot touching the ground with full mass.

The average weight of an adult human being is assumed to be 75 kg. The total weight of clothes, including shoes, is assumed to be an additional 3 kg. The total mass of an operator in an industrial setting is then assumed to be 78 kg. In general, an operator should be able to carry a payload of 20 kg. Considering a minimum weight of 50 kg and a maximum weight of 100 kg per operator we get a range of 50 kg of a light worker without payload and up to 120 kg for a worker with payload. Distributed on two feet, we have a range between 25 kg and 60 kg per foot.

AGVs are available in different sizes and weights. Assuming a standard AGV, we have a total mass between 200 kg up to 1000 kg. Usually the weight is distributed on four wheels. This means a weight per wheel of 50 kg up to 200 kg. As we have an overlapping range of about 50 kg to 60 kg for both, human beings on one foot and AGVs on one wheel, it is not possible to differentiate only by weight. A good overview of the research on pressure sensitive floors can be found in the work of Andries et al. [6]. Other state-of-the-art methods that use pressure-sensitive floors are in [22,23].

### 2.2. Capacitive Sensors

Another possible way to differentiate between human and non-human objects is to measure the change of capacity when an object is approaching a capacitive sensor. There are already sensor systems available that use a capacitive measurement to detect objects in the surroundings of the robot like [7]. The capacity of an object depends on different properties:

- size,
- material,
- humidity, and
- dielectric constant.

Depending on the kind of non-human objects that are present in the application, it could be possible to differentiate between human and non-human objects. AGVs are commonly built with materials like aluminum or steel and have motors and other metallic equipment. For such objects, a capacitive sensor system should be capable of differentiating between human and non-human objects.

Lumelsky et al. were pioneers on the topic of sensitive skin and its use on robot manipulators [24,25]. Other early work like the one from Karlsson and Järrhed [26] proposed one single huge capacitor with one plate on the floor and the second plate on the ceiling above the robot’s workspace. More recent work was done by Lam et al. [27] who managed to integrate the sensors into the housing of the robot manipulator. Thus, reaching a solution where not a single part of the sensor is on the outside of the manipulator that could be damaged.

### 2.3. Thermal Cameras

**Figure 1:**
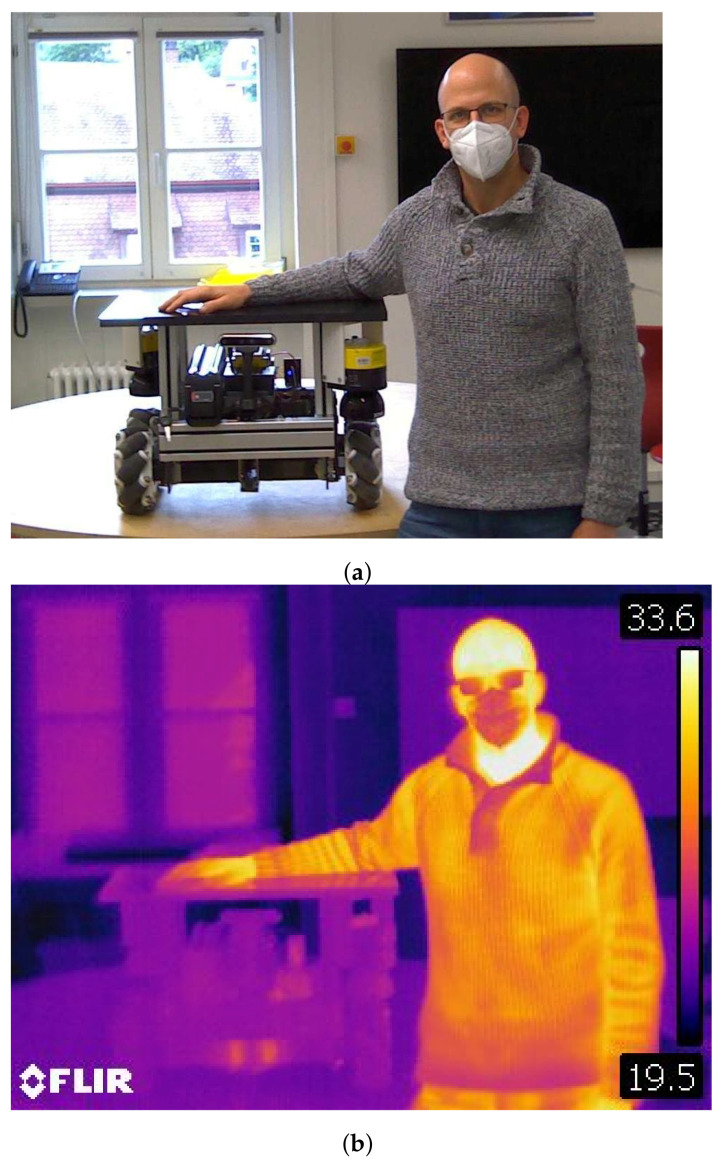
Comparison of visual and thermal image of a human next to an AGV. (**a**) Visual image; (**b**) Thermal image.

Body temperature is a property of a human that is already used in other sensor applications. The human body temperature is usually between 36 ${}^{{}^{\circ}}$C and 37.8 ${}^{{}^{\circ}}$C. There is only a small window allowed for variations. From 37.8 ${}^{{}^{\circ}}$C to 41 ${}^{{}^{\circ}}$C, the human has a physical condition called fever. Above 41 ${}^{{}^{\circ}}$C, the fever can be life-threatening. Everything below 36 ${}^{{}^{\circ}}$C is too cold [28]. Everything above the absolute zero point irradiates infrared light or waves in the infrared spectrum. It can be detected with Bolometers or Thermopiles. In a first measurement, images were taken with a FLIR camera. Note that the human body temperature is only visible on parts of the human that are not covered with clothes or other means of protection like helmets, masks, or safety googles. For the covered parts of the body, the temperature is attenuated, as you can see in Figure 1. Even though the AGV is turned on in the picture, there is no significant heat radiation coming from the AGV next to the human.

### 2.4. Conclusions

There are different sensors that allow a differentiation between human and non-human objects. A differentiation in an industrial setting depends greatly on the conditions in the hall that the system is used in. The decision for a specific sensor needs to made for each individual case. In our work, we continue to focus on the differentiation with thermal cameras.

## 3. Potential Efficiency Improvement

The Cambridge Dictionary defines efficiency as follows: “the good use of time and energy in a way that does not waste any” [29]. For a standard, non-collaborative, robotic application, a common way to measure efficiency is to measure how long the robotic system needs to fulfill a sequence of tasks. With finding ways to shorten the time to fulfill the tasks, one increases the efficiency of the system.

When it comes to human–robot collaborative applications, it gets a bit more complicated. Interactions happen not only with other well-defined objects, but also with a human—and no human is like another. A human in industrial applications is called an operator. This operator might be talking to other operators, might take a break, switch with an other operator, or simply has to clean their nose.

All of these interruptions are not foreseeable for the robotic system and come along with leaving and re-entering the robot’s workspace. The more of these occasions happen, the less efficient is the overall robotic system. An efficient speed and separation monitoring system is essential for these occasions and influences the overall system efficiency.

**Figure 2:**
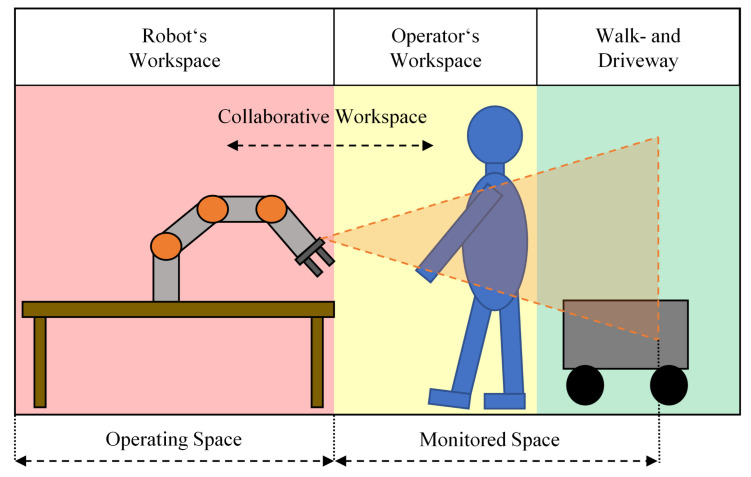
Different workspaces around a robotic application. Note how the monitored space ranges into the walkway and driveway area.

On industrial shop floors, there is usually no hard border for a transition from the walkway or driveway into the operator’s or robot’s workspace as shown in Figure 2. The monitored space can often reach into the walkway and driveways.

In human–robot collaborative applications, there is a special focus on the operator. The safety of the operator has priority over the speed and movement of the robot. This is why the robot has to slow down or come to a complete stop when an operator enters the monitored space. There are different sensor systems that can measure the operators location and speed. So far, these systems do not differentiate between an operator and another machine like an AGV. The AGV is handled like an operator and the robot has to slow down or stop when it gets closer than the protective separation distance.

This is where our work proposes to differentiate between an operator and other machines. This differentiation shall then be taken into account when calculating the protective separation distance. With smaller protective separation distances for non-human objects, we increase the time that the robot can work with higher velocities and thus increase the overall efficiency of the system.

### 3.1. Protective Separation Distance

The point in time when the operator enters the workspace can be variable as well as the speed of the operator while entering the workspace. Depending on the tasks, there are different types and amounts of interaction with other objects. Other objects in this context can be other robots, automated guided vehicles, or human beings. These objects can either provide workpieces, tools, or actively support the robot’s task.

No matter what kind of interaction happens, the object needs to enter and exit the robot’s workspace at a certain point in time. When entering the workspace, the robot has to slow down in order to prevent harm to the object. The moment when to slow down or stop depends on the speed of the robot, the robots reaction and stopping time as well as on the speed of the operator.

This moment is defined as the protective separation distance. The ISO/TS 15066 provides equations to calculate the protective separation distance. This distance depends on a large portion on the operators location and speed. Different values are needed to calculate the protective separation distance. The protective separation distance is calculated as shown in Equation (1) [3]:

(1) $$S_{p}\left(t_{0}\right)=S_{h}+S_{r}+S_{s}+C+Z_{d}+Z_{r}.$$

The different values are defined in the ISO/TS 15066 as follows [3]:

- $S_{p}\left(t_{0}\right)$ is the protective separation distance at time $t_{0}$.
- $t_{0}$ is the present or current time.
- $S_{h}$ is the contribution to the protective separation distance attributable to the operator’s change in location.
- $S_{r}$ is the contribution to the protective separation distance attributable to the robot system’s reaction time.
- $S_{s}$ is the contribution to the protective separation distance due to the robot system’s stopping distance.
- *C* is the intrusion distance, as defined in ISO 13855. This is the distance that a part of the body can intrude into the sensing field before it is detected.
- $Z_{d}$ is the position uncertainty of the operator in the collaborative workspace, as measured by the presence sensing device resulting from the sensing system measurement tolerance.
- $Z_{r}$ is the position uncertainty of the robot system, resulting from the accuracy of the robot position measurement system [3].

The protective separation distance can be a fixed number if worst case values are used to calculate it. Especially the contribution by the human operator plays an important role in the equation.

It is allowed by the ISO/TS 15066 that the protective separation distance can be calculated dynamically according to the robot’s and operator’s speeds [3]. The operators contribution to the overall protective separation distance can be calculated as shown in Equation (2):

(2) $$S_{h}=\;\int_{t_{0}}^{t_{0}+T_{r}+T_{s}}v_{h}\left(t\right)\;dt.\;$$

A constant value for $S_{h}$ can be calculated with Equation (3):

(3) $$S_{h}=1.6\cdot T_{r}+T_{s}.$$

Equation (4) shows how to calculate the distance that the robot moves during the reaction time of the controller of the robot:

(4) $$S_{r}=\;\int_{t_{0}}^{t_{0}+T_{r}}v_{r}\left(t\right)\;dt.\;$$

A constant value for $S_{r}$ can be calculated with Equation (5):

(5) $$S_{r}=v_{r}\left(t_{0}\right)\cdot T_{r}.$$

The contribution of the stopping time can be calculated with Equation (6):

(6) $$S_{s}=\;\int_{t_{0}+T_{r}}^{t_{0}+T_{r}+T_{s}}v_{s}\left(t\right)\;dt.\;$$

### 3.2. Object-Specific Protective Separation Distance

Our proposal in this paper is to introduce an additional variable in the formula for the protective separation distance for the object kind. There are two different approaches of how to handle this additional variable.

One is to treat the variable as a binary digit: the value is either 0 or 1. If the object is a human, the contribution of the operator’s change in location to the protective separation distance needs to be fully accounted for and the value is set to 1. If the object is a non-human object, the variable is set to 0 and the contribution of the object to the protective separation distance is neglected.

The second approach would be to treat the value as a probability of how likely the object is a human or a non-human object. With 0 being a non-human object and 1 being a human. Equation (7) shows the formula for the extended protective separation distance:

(7) $$S_{p}\left(t_{0}\right)=\left(S_{h}\cdot T\right)+S_{r}+S_{s}+C+Z_{d}+Z_{r}.$$

In order to get a rough estimate of values for the protective separation distance, we calculate an example for the protective separation distance. We calculate with $v_{r}=2.5\;m/s$ and an operator velocity of 1.6m/s.

The specification sheet for the KUKA LBR iiwa 7 R800 specifies a stopping distance of 5.193${}^{{}^{\circ}}$ for a category 0 Stop on axis 1 with a 100% radius and a 100% program override. With a specified radius of 800 mm for the KUKA robot, the distance traveled during stopping would be 72.47 mm according to Equation (8):

(8) $$S_{s}=2\cdot \pi \cdot 800\;\mathrm{mm}\cdot \frac{5.193^{{}^{\circ}}}{360^{{}^{\circ}}}=72.47\;\mathrm{mm}.$$

**Figure 3:**
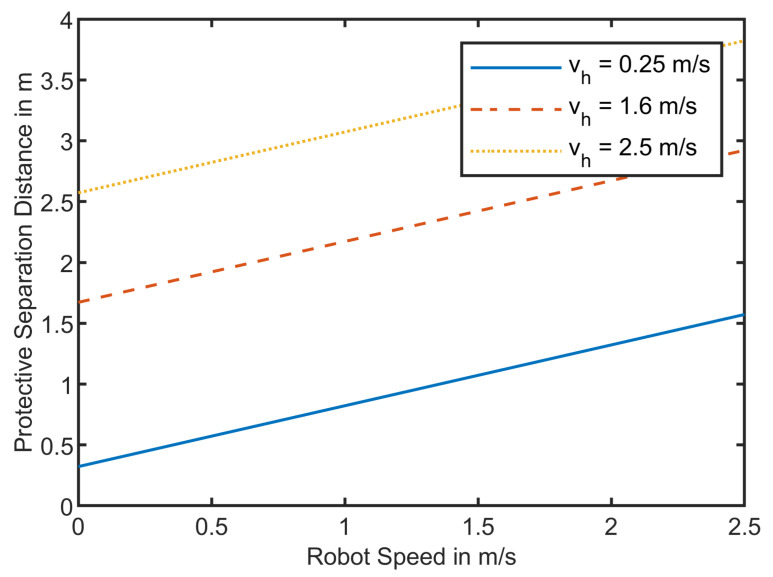
Protective Separation Distances for robot speeds between 0 m/s and 2.5 m/s and operator speeds of 0.25 m/s, 1.6 m/s, and 2.5 m/s.

Neglecting the values for position uncertainties of the robot and the operator, and neglecting the intrusion distance, we can plot the protective separation distance for robot speed of 0 to 2.5 m/s with operator speeds of 0.25 m/s which is the maximum allowed speed close to the robot, 1.6 m/s as an average operator speed, and 2.5 m/s as maximum speed. Figure 3 shows the calculated protective separation distances. The protective separation distance is linearly dependent on the robot and the human velocity. If the robot moves with full speed of 2.5 m/s and the operator approaches the system with a speed of 1.6 m/s, the protective separation distance is 2.922 m.

Figure 3 shows the dependency of the robot’s and the operator’s speed on the protective separation distance. If it is possible to differentiate between an operator and an AGV, there would be no need for accounting for the approaching distance of the operator and $S_{h}$ could be neglected. This reduces the protective separation distance for a robot’s speed of $v_{r}=2.5$ m/s from 3.5 m down to 1.5 m. This opens a range of 2 m where the AGV can drive by the robotic system without interfering with the robot’s speed.

## 4. Measurements

### 4.1. Monitored Space

A difficult question is always what needs to be monitored by the sensors system. Typically, a robot’s workspace is divided into two main sections, as shown in Figure 2, namely, the operating space and the collaborative workspace. The collaborative workspace is the part where the operator can work collaboratively with the robot. The operating space is the part where no human being is allowed and where the robot can work faster than in the collaborative workspace.

Considering a robot that is capable of moving 360${}^{{}^{\circ}}$ around its base, the collaborative workspace can be as small as a few degrees or as big as the full 360${}^{{}^{\circ}}$ around the base. Thus, the size of the collaborative workspace is calculated as follows:

(9) $$Size\;of\;Collaborative\;Workspace=360^{{}^{\circ}}-Size\;of\;operating\;space.$$

The operating space is protected by design against any access of the operator. The collaborative workspace needs to be monitored with a sensor system that is capable of measuring the separation distance to an intruding obstacle like the operator or an AGV.

A sensor for monitoring the collaborative workspace has a defined field of view (FoV). The amount of sensors needed to monitor the entire collaborative workspace is then calculated as shown in Equation (10):

(10) $$Number\;of\;sensors\;needed=\frac{Size\;of\;Collaborative\;Workspace}{FoV}.$$

The collaborative workspace ends with the maximum reach of the manipulator. In order to calculate the protective separation distance we need to be able to detect obstacles before they enter the collaborative workspace.

Therefore, monitoring is necessary for the collaborative workspace and an additional extended monitoring space. This extended monitoring space usually includes walkways for other workers and AGVs. The required size of the extended monitoring space must be at least the maximum possible protective separation distance as calculated in Section 3. The sensor for differentiating between human and non-human objects must have the same range.

As seen in Figure 3, the maximum possible protective separation distance for robot speed of $v_{r}=2.5\;m/s$ and an operator speed of $v_{h}=2.5\;m/s$, is $S_{p,2.5}=3.822\;m$. We round up and set the maximum separation distance to $S_{p,max}=4\;m$.

The goal is to be able to detect a temperature of a human being in an industrial surrounding in a distance of $S_{p,max}=4\;m$.

As described in Section 2, the human body temperature can usually only be measured somewhere in the head area of the operator due to clothing covering the skin of the rest of the body. Let us assume a head size of an average human being of 20 cm. We want to be able to have a minimum pixel size for measurement of 10 cm in a distance of $S_{p,max}=4\;m$. The pixel size in different distances from a sensor is calculated as shown in Equation (11):

(11) $$x=2\tan\left(\frac{\alpha }{2}\right)d.$$

**Figure 4:**
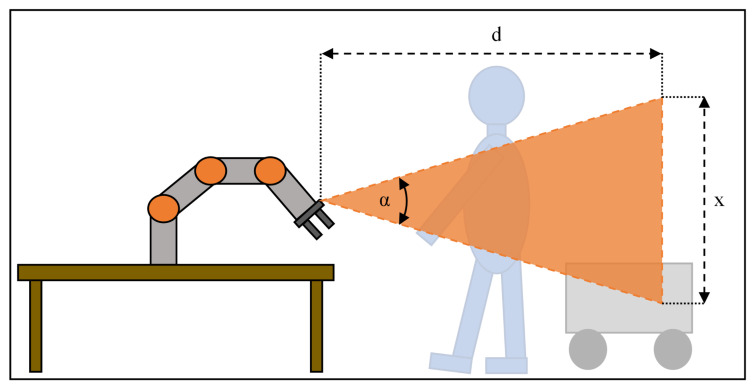
Schematic for the field of view of the sensor attached to the flange of the robot.

With *d* being the distance from the sensor to the object, α the field of view of the sensor, and *x* the size of the viewing window in a distance *d* from the sensor as shown in Figure 4.

The commercially available TeraRanger Evo Thermal 33 and Evo Thermal 90 are used to make measurements. The properties of the sensors are listed in Table 3. The sensor is connected via USB to a laptop running Windows 10. Matlab is used to read the data from the sensor via a serial connection with parameters set to: Baud Rate of 115,200, 8 Data Bits, 1 Stop Bit, Parity None, and no flow control. Matlab was chosen due to its great ability to work with matrices as the data read from the sensor with its resolution of 32 × 32 pixels is best represented in a 32 × 32 matrix. Furthermore, Matlab provides a well-established set of functions for postprocessing the data. With the KUKA Sunrise Toolbox it is possible to control the KUKA LBR iiwa 7 R800 robot directly with Matlab via an Ethernet connection [30]. This allows the control of the entire measurement setup with only one laptop running Matlab.

**Figure 5:**
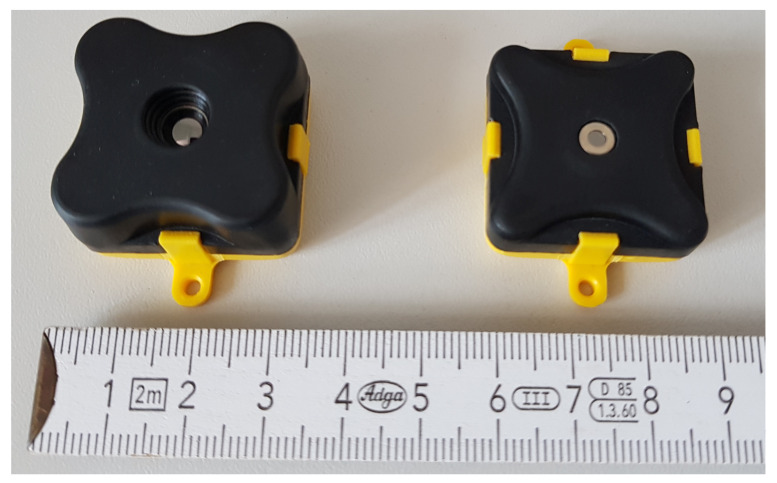
Teraranger Thermal 33 and 90.

The two sensors from Terabee have a field of view of 90${}^{{}^{\circ}}$ and 33${}^{{}^{\circ}}$. The sensors are shown in Figure 5. The resolution is 32 × 32 pixels. The size of the area measured by the sensor in a distance *d* is calculated by dividing Equation (11) by 32 pixels as shown in Equation (12):

(12) $$x_{Sensor}=\frac{2\tan\left(\frac{\alpha }{2}\right)d}{32}.$$

**Figure 6:**
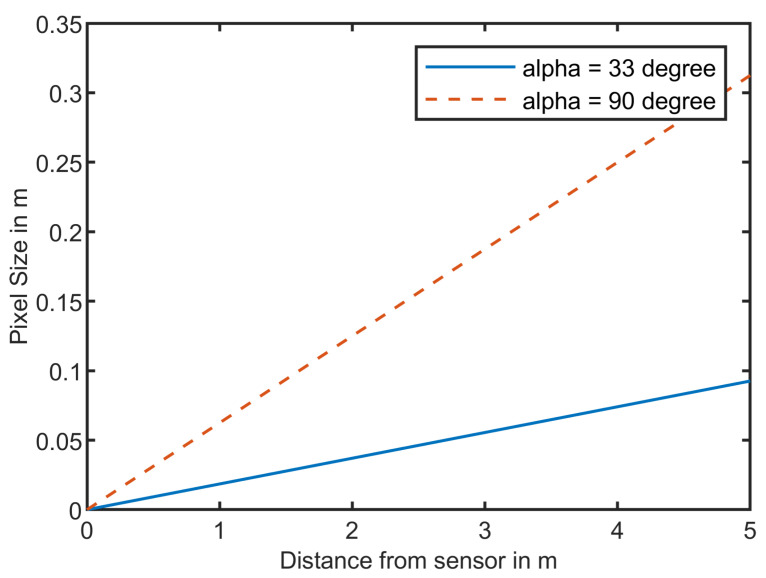
Size of pixel in different distances from the sensor.

The pixel sizes for both sensors for distances from 1 m up to 5 m are shown in Figure 6.

The average size of a human head is assumed to be 20 cm. The pixel size of the 33${}^{{}^{\circ}}$ FoV sensor in a distance of 5 m is ~10 cm according to Equation (11). For the 90${}^{{}^{\circ}}$ FoV sensor, the pixel size would already be at 30 cm in a distance of 5 m which would not lead to good results. A pixel size of 10 cm for the 90${}^{{}^{\circ}}$ FoV sensor is reached at a distance of 2 m.

A first measurement was to see if it is possible to measure the human temperature in different distances of 1 m to 4 m in 1 m steps. With Matlab, the average temperature of 10 subsequent measurements was calculated and plotted in an thermal image. The room temperature during the measurement was 22.2 ${}^{{}^{\circ}}$C and the humidity was at 56%.

In order to find out if it is possible to detect an operator within 4 m from the robot, we make following measurement. The sensor is placed in a height of 120 cm. The sensor is connected via USB to a laptop running Windows 10 and Matlab. Matlab opens a serial connection to the sensor. The Matlab script reads the temperature values from the sensor 100 times. In a first measurement, there is no operator or other human being in the field of view of the sensor. In the next eight measurements, there is an operator with a height of 183 cm in distances of 0.5 m to 4 m in 0.5 m steps. At each distance value, 100 measurements are taken. Matlab then calculates the mean value for each measurement as well as the standard deviation. This measurement will show if it is possible to see the difference between human beings and the surroundings.

### 4.2. Differentiation Algorithm

**Figure 7:**
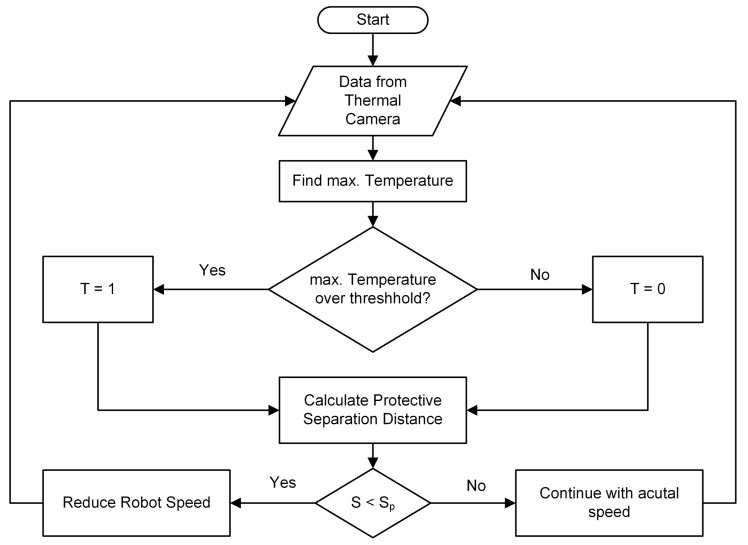
Flow chart of temperature decision for speed adaption.

In order to save computing time, the first approach is to measure the temperature and compare it to a threshold as shown in the flow chart in Figure 7.

First, the thermal data from the camera are read via a serial connection. Second, the Matlab function *max*() is used to find the maximum measured value. Third, the measured maximum temperature is compared with a threshold. If the maximum measured temperature exceeds the threshold, the variable T is set to 1, meaning that the object is treated like a human. If the measured temperature stays below the threshold, the variable T is set to 0, meaning that there is no human in the field of view of the sensor and that the object must be a machine. Fourth, the extended protective separation distance as introduced in Section 3 is calculated. In the last step, the robot’s speed is adjusted according to the calculated extended protective separation distance.

The temperature threshold needs to be set depending on the application. Best results will be achieved in settings where the temperature of the surrounding equipment is significantly lower than the temperature of a human being. With typical room temperatures of less than 23 ${}^{{}^{\circ}}$C, a threshold for the measurements of 24 ${}^{{}^{\circ}}$C is chosen.

## 5. Results and Discussion

**Figure 8:**
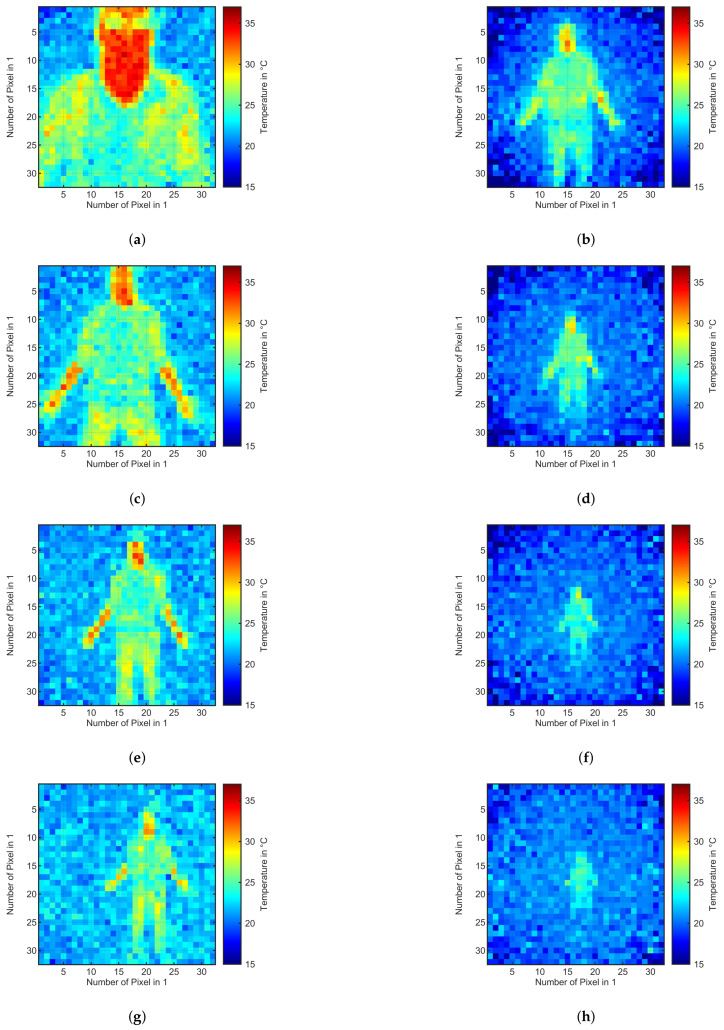
Thermal images of human in distances of 1 m, 2 m, 3 m, and 4 m of the two sensors TeraRanger Evo Thermal 33 and 90. (**a**) Human in 1 m distance of Evo Thermal 33; (**b**) Human in 1 m distance of Evo Thermal 90; (**c**) Human in 2 m distance of Evo Thermal 33; (**d**) Human in 2 m distance of Evo Thermal 90; (**e**) Human in 3 m distance of Evo Thermal 33; (**f**) Human in 3 m distance of Evo Thermal 90; (**g**) Human in 4 m distance of Evo Thermal 33; (h) Human in 4 m distance of Evo Thermal 90.

Figure 8 shows the eight results for the thermal measurements of both sensors. Figure 8a,c,e,g show the results with the TeraRanger Evo Thermal 33. As seen in Figure 8a, the human temperature is measured quite well with a mean temperature over 10 measurements of 34.92 ${}^{{}^{\circ}}$C. In Figure 8a, one can also see that the human is wearing glasses. Glasses have poor transmission of long-wave infrared radiation and therefore we see a lower temperature on the glasses. This could be a possible solution for AGVs that show a certain heat radiation from their motors or electronics. Those parts could be covered by a glass or another material that does not transmit heat radiation. In Figure 8c,e,g, you can see that the underarms of the human being were not covered and therefore also were measured with a temperature in the range of 30 ${}^{{}^{\circ}}$C.

Figure 8b,d,f,h show the four results for the thermal measurement of a human-being in distances of 1 m to 4 m in 1 m steps with the Terabee Evo Thermal 90 sensor. Figure 8b shows that the bigger FoV of 90${}^{{}^{\circ}}$ allows to measure almost a complete standing operator in a short distance of only 1 m compared to only half the operator in Figure 8a. As calculated in Section 4, you can see that in Figure 8f,h the operator and especially the head are so far away, that one pixels measures more than just the temperature of the head. This leads to a significantly reduced average temperature. That makes it harder to differentiate the operator from its surroundings.

Figure 8 shows two main advantages and drawbacks of the sensors. For the Evo Thermal 33 sensor, the main drawback is the small field of view. Depending on the application, multiple sensors might be needed to cover the entire area that needs to monitored. The advantage is that the measured temperature is close to actual temperature for the entire distance range from 1 m up to 4 m. This is the drawback of the Evo Thermal 90 sensor, that still measures temperatures over 30 ${}^{{}^{\circ}}$C for distances up to 2 m. However, for distances above 2 m, the single pixels of the sensor cover areas of 12.5 cm by 12.5 cm and more, resulting in lower temperature measurements if a body part only covers a part of the pixel. Depending on the room temperature it gets more and more difficult to detect a human being in distances of more than 2 m for the Evo Thermal 90 sensor. The advantage of the Evo Thermal 90 sensor is the field of view that allows to cover a three times bigger area than the Evo Thermal 33.

**Figure 9:**
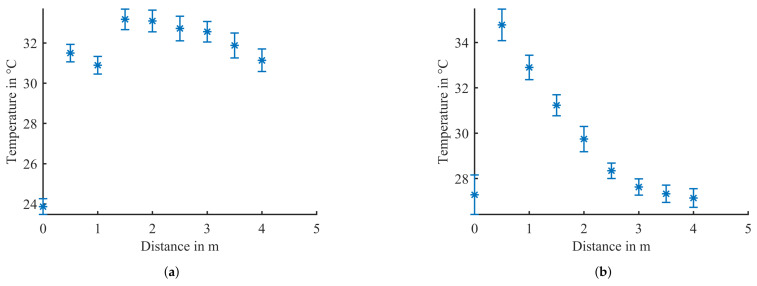
100 Measurements with human-being in distances from 0.5 m to 4 m for both sensors, the TeraRanger Evo Thermal 33 and 90. (**a**) Evo Thermal 33: 100 Measurements with human in distances from 0.5 m to 4 m; (**b**) Evo Thermal 90: 100 Measurements with human in distances from 0.5 m to 4 m.

Figure 9 shows the results of the measurement where the highest temperature was measured while an operator was in distances of 0.5 m to 4 m in 0.5 m steps from the sensor. The measurement was executed once with the Evo Thermal 33 and once with the Evo Thermal 90. For each distance of the operator, 100 measurements were taken. The mean value was calculated and plotted in Figure 9 with error bars for the standard deviation. The record for a distance of 0 m represents the measurement without operator in the field of view of the sensors.

Figure 9a shows that for the Evo Thermal 33 sensor, there is a difference of more than 5 ${}^{{}^{\circ}}$C between the temperature measurements in all different distances compared to the temperatures measured without an operator present.

The lower mean values for distances 0.5 m and 1 m with the Evo Thermal 33 as shown in Figure 9a can be explained by the narrow field of view of the sensor. Due to the sensor being placed in a height of 120 cm and the FoV being 33 ${}^{{}^{\circ}}$, the sensor cannot measure the temperatures from the head of a 183 cm operator. Due to the operator wearing long-sleeved shirt, the mean values are a bit lower because the sensor does not see any naked skin that would radiate more heat. Starting at a distance of 1.5 m, the head of the operator with a lot of exposed skin is lying in the field of view of the sensor and therefore detected with a higher mean temperature than the measurements of 0.5 m and 1.0 m.

Figure 9b shows that the measurement for the scene without an operator shows a similar temperature range like the temperatures measured in distances of 2.5 m and more. Therefore, it will not be possible to make a differentiation between human and non-human objects with the Evo Thermal 90 sensor in distances above 2 m. This confirms the result of Figure 8 and is one of the main drawbacks of the Evo Thermal 90 sensor.

Regarding the proposed algorithm, these results show that for normal room temperatures below 24 ${}^{{}^{\circ}}$C, it is possible to make a differentiation between human beings and other machines like AGVs. One drawback is in case that an AGV exposes a heat source like a motor or an electric device that radiates heat in the same amount like a human being, the AGV could be mistakenly be treated like an operator. This might lead to a reduced efficiency, but it would not be a safety issue for the operator. A possible solution would be to cover the heat source with a material that does not allow transmission of infrared heat. The main advantage of this algorithm is its simple structure and therefore short computing time.

An interesting question arises when looking at the corona pandemic where one main indicator for human health is body temperature. Pictures on TV showed that people had their temperature measured on their forehead, a region that is also part of the measurement in our setup. Considering the entire possible temperature range of a human being between 36 ${}^{{}^{\circ}}$C and 41 ${}^{{}^{\circ}}$C, this should not affect the system performance. For setups where the human is the warmest object, it is no problem at all. The threshold will be set depending on the room temperature and the given temperatures of the surroundings. Everything above that temperature will be treated as a human being. It will become more important in setups where the system should be able to differentiate a human from objects that are warmer than the human. If the object’s mean temperature is close to 41 ${}^{{}^{\circ}}$C, then it will be difficult to make a correct differentiation. The differentiation will be easier when the object’s temperature is essentially higher than the human’s core temperature.

## 6. Conclusions and Outlook

This paper introduced an object-specific protective separation distance for speed and separation monitoring in human–robot collaborative applications. The use case was that in small- and medium-sized enterprises the shop floor space is limited. The space that needs to be monitored for speed and separation monitoring in HRC applications overlap with the walkways and driveways for other operators and AGVs. AGVs that pass through this monitored space slow down the robotic applications because they are treated like an operator. Differentiating between operators and AGVs allows to adjust the protective separation distance and therefore let the robot move with higher speed.

The main feature that differentiates an operator from an AGV is its temperature. Using a thermal camera, it is possible to differentiate between a human and an AGV in distances of up to four meters depending on the resolution and on the field of view of the sensor. The measurements showed that the smaller FoV sensor has advantage in measuring the temperature of objects in distances of 2 m and more. The 90${}^{{}^{\circ}}$ FoV sensor had the advantage of being able to measure the entire height of an operator in distances as close as 1 m. A mix of both sensors will be subject for further research. A disadvantage of this method is that if the AGV exposes a heat source like a motor or an other electric device, it can mistakenly be treated as an operator. In these cases, the heat sources on the AGV must be covered.

The paper showed that there is potential of more than 50% to decrease the protective separation distance and therefore increase the efficiency of the overall collaborative robotic system. The object-specific protective separation distance differentiates between human and non-human objects in the vicinity of the robot’s workspace through the use of thermal cameras.

The proposed differentiation between human and non-human objects might not only be beneficial for Speed and Separation Monitoring, but also for power- and force-limiting operations. The power- and force-limiting operation is based on maximum values for quasi-static and transient contacts [3]. The values are determined in a risk assessment for the specific application.

Similar to the situation in speed and separation monitoring, there is no need to treat non-human objects like a human object. For hon-human objects, the maximum values for quasi-static and transient contacts can be higher. The amount of how much higher these values can be set depends on the materials that the non-human objects are made of. With a sensor system that can differentiate between human and non-human objects, it is possible to adjust the maximum values for the power and force limiting operation. The robot will be able to move with higher speed when a non-human object is close by and therefor the overall efficiency will be increased. This topic will be subject for further investigation.

Furthermore, research in the future will investigate the different presented sensor systems and how well they are suitable for human–machine differentiation. Fusing the data of different sensors might lead to even better results. A first step will be to combine an infrared ToF sensor with the thermal camera in order to get a single sensor system. Another important task is to look at how the different sensor systems can be compared and how the overall system efficiency can be described to suit a broader spectrum of applications. 

## Figures and Tables

**Table 1:** List of main contributions of this paper.

Main Contributions
Introduction of human–machine differentiation into speed and separation monitoring.
Introduction of the extended protective separation distance formula that is extended with a variable for human and non-human objects.
An algorithm for detecting an operator in distances of up to 4 m with thermal cameras directly from the manipulator.
Experimental verification of the algorithm with two thermal cameras with different field of views.

**Table 2:** Overview of active and passive methods for human–machine differentiation.

Active Methods	Passive Methods
Wireless Transmission of position	Temperature
Camera and QR-codes	Heart Beat
Camera and Object Recognition	Breath
Sound localization	Walking Pattern
	Dielectric constant

**Table 3:** Teraranger Evo Thermal Specifications [31].

Specification	Evo Thermal 90	Evo Thermal 33
Resolution	32 × 32 pixels	32 × 32 pixels
Field of View	90${}^{{}^{\circ}}$ × 90${}^{{}^{\circ}}$	33${}^{{}^{\circ}}$ × 33${}^{{}^{\circ}}$
Temperature Range	−20 ${}^{{}^{\circ}}$C to 670 ${}^{{}^{\circ}}$C	30 ${}^{{}^{\circ}}$C to 45 ${}^{{}^{\circ}}$C
Update Rate	7 Hz	7 Hz
Range	up to 5 m	up to 5 m
Size	29 × 29 × 13 mm	29 × 29 × 22 mm
Weight	10 g	12 g

## Data Availability

Measurement data is available on request from the corresponding author.
